# Experimental Study on the Performance of Steel-Fiber-Reinforced Concrete for Remote-Pumping Construction

**DOI:** 10.3390/ma16103666

**Published:** 2023-05-11

**Authors:** Minglei Zhao, Changyong Li, Jie Li, Lixian Yue

**Affiliations:** 1 School of Engineering, RMIT University, Melbourne 3001, Australia; s3339909@student.rmit.edu.au; 2 International Joint Research Lab for Eco-Building Materials and Engineering, North China University of Water Resources and Electric Power, Zhengzhou 450045, China; z201910311304@stu.ncwu.edu.cn

**Keywords:** steel-fiber-reinforced concrete, remote pumping, mix proportion, pumpability, rheology, strength, impact resistance, water impermeability

## Abstract

Remote-pumped concrete for infrastructure construction is a key innovation of the mechanized and intelligent construction technology. This has brought steel-fiber-reinforced concrete (SFRC) into undergoing various developments, from conventional flowability to high pumpability with low-carbon features. In this regard, an experimental study on the mixing proportion design and the pumpability and mechanical properties of SFRC was conducted for remote pumping. Using the absolute volume method based on the steel-fiber-aggregate skeleton packing test, the water dosage and the sand ratio were adjusted with an experimental study on reference concrete with the premise of varying the volume fraction of steel fiber from 0.4% to 1.2%. The test results of the pumpability of fresh SFRC indicated that the pressure bleeding rate and the static segregation rate were not the controlling indices due to the fact that they were far below the limits of the specifications, and the slump flowability fitted for remote-pumping construction was verified by a lab pumping test. Although the rheological properties of the SFRC charactered by the yield stress and the plastic viscosity increased with the volume fraction of steel fiber, those of mortar used as a lubricating layer during the pumping was almost constant. The cubic compressive strength of the SFRC had a tendency to increase with the volume fraction of steel fiber. The reinforcement effect of steel fiber on the splitting tensile strength of the SFRC was similar to the specifications, while its effect on the flexural strength was higher than the specifications due to the special feature of steel fibers distributed along the longitudinal direction of the beam specimens. The SFRC had excellent impact resistance with an increased volume fraction of steel fiber and presented acceptable water impermeability.

## 1. Introduction

With the mechanized and intelligent development of concrete technology, ready-mixed concrete produced in factories, transported by a concrete-mixing carrier, and placed in situ by pumping equipment has become a common concrete-placing method [1,2,3]. Furthermore, ready-mixed concrete directly pumped in a pipeline over super-high and long distances provides a great technological advantage with a much quicker delivery [2,3,4]. This creates the possibility of remotely pumping concrete for constructing high-rise buildings, long-span and overlength bridges, and long tunnels with concrete linings. In this regard, remote-pumped concrete has the benefits of further improving construction efficiency and saving on concrete placing and labor costs due to the fact that it overcomes the difficulty of in situ vehicle transportation and belt transmission limited by site conditions, it simplifies the construction site layout without the requirements of road laying for concrete mixing carriers, and it reduces the in situ interference of construction machinery and equipment [5,6,7,8]. Up to now, the pumping height of concrete is limited to about 200 m while the pumping distance is about 300 m. However, more and more attempts have been attempted to break these records [8,9,10]. In recent years in China, about 30 ultra-high buildings over 300 m in height have been built or are being built with pumped concrete. For instance, the Shanghai World Financial Center built in Shanghai is 97 floors high with a structural height of 492 m, and the pumping height of the concrete reached 492 m for constructing the reinforced concrete core tube. The Tianjin 117 building built in Tianjin is 117 floors high with a structural height of 597 m, and the pumping height of the concrete reached 621 m for constructing the giant reinforced concrete column structures [10,11]. In addition, the long-distance pumping of concrete has also been used for constructing overlength bridges. For instance, the Zangmu Yarlung Zangbo river bridge of the Sichuan–Tibet Railway is a concrete-filled steel-tube half-through arch bridge with a 430 m long span and a prestressed concrete box beam of 518.8 m in length; the pumping distance of the concrete was 550 m for the bridge construction [8,12].

Indeed, there are lots of challenges in the development of pumped concrete, including the proper selection of pumping equipment that can supply a sufficient pump pressure with a continuously controllable flow of concrete in the pipeline, suitable pumping pipes that depend on the practical conditions, the required flow rate and the pumping pressure, and a pumpable concrete that influences the pumping pressure with a rational lubrication layer between the pumped concrete and inner face of pump pipe [9,10,13,14,15]. Among these, the pumpability of pumped concrete plays a dominant role in successfully completing a pumping construction that avoids the blocking or blowouts of the pump or pipeline. Therefore, many studies have been carried out on the mix proportion, the fresh properties before and after pumping, and the flowability during the pumping of the pumped concrete. Generally, a greater flowability with a steady and homogenous composition without water bleeding is a basic requirement of remote-pumped concrete [2,3,16]. To achieve this target, an issue that should be solved first is the synergistic design of the mix proportion of remote-pumped concrete, which is influenced by many factors. Except for admixing the mineral admixtures such as fly-ash, silica fume, ground-granulated blast slag powder, and superplasticizers such as high-performance water reducers and compound pumping agents, the flowability of pumped concrete can be improved by reducing the dosage and particle size of the coarse aggregate [10,16,17,18]. Mixing with a proper content of super-fine river sand and coarse manufactured sand provides the benefits of improving the flowability of concrete due to the round grains of super-fine river sand making up for the deficiencies of coarse manufactured sand with its rough and irregular shape [4,13]. More importantly, a sufficient amount of mortar in concrete facilitates a concrete flow that forms a lubrication layer near the inner surface of the pump pipe during the pumping of concrete due to shear-induced particle migration. The lubrication layer is mainly composed of a binder paste with a lower quantity of sand particles, which has the best rheological properties with a lower viscosity and yield stress. This is indispensable for markedly decreasing the friction of the flowing concrete on the pipe wall [10,14,15,18,19]. Compared to the height of vertically pumped concrete, the distance of horizontally pumped concrete can be converted with a ratio of 3, 4, or 5 for a pump pipe with diameter of 100 mm, 125 mm, or 150 mm, respectively [4]. However, a study conducted by Kim et al. [20] revealed that the ratio of the horizontal distance to the vertical height of pumped concrete decreased with the increase in the plastic viscosity of the lubrication layer.

Comparatively, pumped concrete with a lower dosage and particle size of coarse aggregate has a tendency to reduce the cracking resistance of concrete at an early age or under load with a greater shrinkage. This can be overcome by adding steel fibers that are randomly distributed in the concrete matrix. With the effects of confinement to deformation and bridging the micro- and macro-cracks of the concrete matrix, steel-fiber-reinforced concrete (SFRC) presents a specific performance that relates to the tensile properties, including a decreased shrinkage, especially at an early age, a strengthened freeze–thaw durability, an enhanced tensile strength and flexural toughness, and an improved impact resistance [21,22]. However, most of the studies on this type of concrete have concentrated on the plastic SFRC and flowability SFRC as well as self-compacting SFRC [23,24,25], while a few of these studies were concerned about SFRC for common pumping heights/distances by truck pump [26,27]. Up to now, few of them have been conducted concerning the pumpability and related hardened properties of SFRC for remote-pumping construction. Jiang et al. [28,29,30] reported that by using crushed basalt stones with a maximum particle size of 20 mm and shear-cut indented steel fibers with a length of 20 mm, SFRC could be designed for vertical pumping up to 308 m with the required pumpability, although the experimental pressure bleeding rate was 36%, which was close to the limit of 40% specified in the Chinese code JGJ/T 10 for pumped concrete [5]; the flexural toughness of the hardened SFRC was markedly improved, with the initial and ultimate flexural strengths being increased by 15.6% and 31.4%, respectively, the structural crack width was decreased by 30%, and the drying shrinkage was reduced by 50%, while a better fatigue resistance, freeze–thaw durability, and impermeability were presented.

Obviously, the limited studies in previous reports are insufficient to develop a mature remote-pumping technology for FRC. Therefore, a deep study is necessary to make use of the outstanding performance of pumped SFRC. In this paper, a plan was made to propose a suitable design method for the mixing proportion and to determine the necessary indices representing the pumpability of SFRC for remote-pumping construction. From the point of the design requirements, the basic mechanical properties, including the cubic compressive strength that represents the strength grade, and the splitting tensile and flexural strengths that reflect the reinforcing effect of the steel fibers, were experimentally studied. In addition, important performance metrics, including the impact resistance and the water penetration of the remote-pumped SFRC, were also examined. The effects of steel fiber were analyzed based on the test results and compared with those specified in the codes for conventional SFRC.

## 2. Materials and Methods

### 2.1. Raw Materials

Ordinary silicate cement with a strength grade of 42.5 produced by Xinxiang Tianrui Cement Co. Ltd., Henan, China, was used with the properties summarized in Table 1, which met the requirement of the Chinese code GB 175 [31]. Low-calcium class-I fly-ash was supplied by Hebi Tongli Building Materials CO., Ltd., Henan, China, and was used as the mineral admixture with the properties summarized in Table 2, which met the requirements of the Chinese codes GB/T 1596 [32]. As summarized in Table 3, which summarizes the chemical compositions of the cement and fly-ash, the high contents of the active chemical composition of SiO_2_ and Al_2_O_3_ of fly-ash with a lot of nanoscale spheroidal particles contributed to achieving a secondary hydration with the cement hydrate Ca(OH)_2_, which was accompanied by the tiny aggregate action and the enhancing of the interfacial transition zone, thus improving the properties of concrete [33,34].

Ingot-milled steel fiber, a kind of formalized steel fiber produced by a specialized machine, provided by Shanghai Harix Steel Fiber Technology CO., Ltd., Shanghai, China, was used with a length *l*_f_ = 32.6 mm, an equivalent diameter *d*_f_ = 1.1 mm, and a tensile strength over 860 MPa. The steel fibers is rational to well distribute in concrete matrix [35].

To obtain a sufficient flow of coarse aggregate in the fresh mix, the size of the coarse aggregate used for pumped SFRC should be limited. The maximum particle size of the coarse aggregate should be less than 20 mm for a pipe diameter of 100 mm [5], and it should be less than 3/4 of the length of the steel fiber. Therefore, the maximum particle size was determined as 20 mm in this study [35]. Crushed limestone was mixed with 5–10 mm and 10–20 mm particles to meet the continuous gradation specified in the Chinese code JGJ 52 [36]. Natural river sand with a fineness modulus of 2.65 was gathered from the beach of Tanghe River, Henan, China. The main properties of the aggregates are summarized in Table 4.

**Figure 1:**
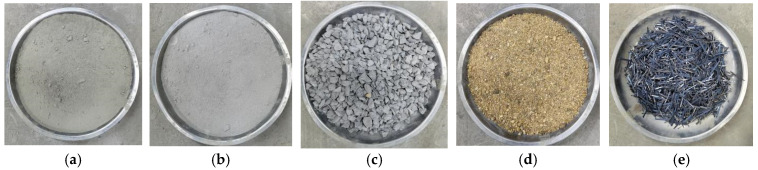
Pictures of the main raw materials: (**a**) cement; (**b**) fly-ash; (**c**) crushed limestone; (**d**) river sand; (**e**) steel fibers.

Pictures of the main raw materials including cement, fly-ash, crushed limestone, river sand, and steel fibers are exhibited in Figure 1.

The additive was selected based on fluidity tests of the cement paste [37]. The compound pumping agent with a water reduction of 23.7% produced by Jiangsu Subot New Materials Co. ltd., Nanjing, China, was used as a superplasticizer in this study.

### 2.2. Mix Proportion

The design of the mixing proportion was carried out concerning the proper pumpability of concrete for remote-pumping construction with a target compressive strength of 48.2 MPa for a concrete strength grade of C40 [38]. According to the Chinese code JGJ/T 10 for pumped-concrete construction, the horizontal pumping distance can be converted from the vertical pumping height by considering the pump pipe diameter that relates to the particle size of the coarse aggregate [5]. With a pipe diameter of 100 mm, the flowability of pumped concrete with the pumping height/distance are presented in Table 5.

**Figure 2:**
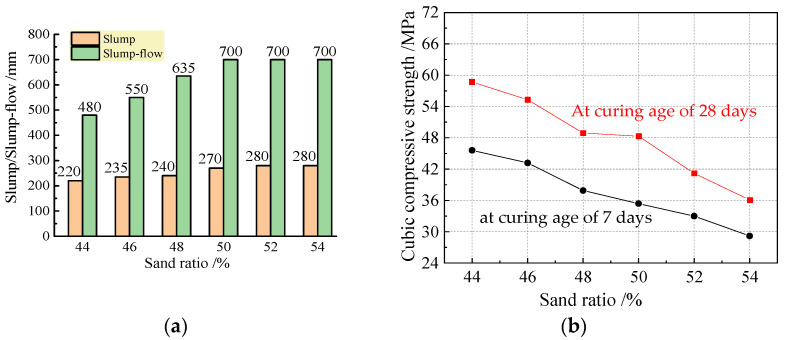
Influence of sand ratio on concrete: (**a**) flowability; (**b**) cubic compressive strength.

The mixing proportion of the reference concrete was firstly designed to provide a basis for the design of the remote-pumping SFRC using the absolute volume method [38,39]. A fly-ash content of 30% was selected accounting for its influence on the compressive strength of cement, and the water-to-binder ratio was calculated to be 0.35. With the premise of a target flowability of the fresh mix that met the requirements of remote pumping over 1200 m as listed in Table 5, the water dosage was taken as 178.1 kg/m^3^, and the compound pumping agent dosage was determined as 1.4% of the total mass of the binders. The sand ratio varied from 44% to 54% based on the closing packing test. According to the specifications in the Chinese codes GB 50080 and GB 50081 [40,41], the test results of the flowability of the fresh mix and the cubic compressive strength of the concrete with different sand ratios are summarized in Figure 2. The fresh mix with a sand ratio of 44% was viscous, and that with a sand ratio of 54% was segregated with water bleeding. The flowability of the fresh mix increased with a sand ratio that was not over 50%, and the cubic compressive strength obviously decreased with the increase in the sand ratio. Combined with the concrete that reached the target cubic compressive strength of 48.2 MPa, the sand ratio was not over 50%.

The mixing proportion of the remote-pumping SFRC was designed with reference to the absolute volume method based on the steel-fiber–aggregate skeleton packing test [42]. Based on the tests of the reference concrete, the water dosage and the sand ratio of the remote-pumping SFRC were adjusted by the following formulas:

(1) $$m_{w,f}=\left(1+0.25F\right)m_{w,0}$$

(2) $$\beta_{s,f}=\left(1+0.11F\right)\beta_{s,0}$$

(3) $$F=\left(\frac{l_{f}}{d_{f}}\right)v_{f}\eta_{f}$$

where *m*_w,f_ and *β*_s,f_ are the water dosage and the sand ratio of the remote-pumping SFRC, respectively; *m*_w,0_ and *β*_s,0_ are the water dosage and the sand ratio of the reference concrete, respectively; *F* is the fiber factor; *v*_f_ is the volume fraction of steel fiber; and *η*_f_ = 0.90 for the ingot-milled steel fiber.

Finally, the mixing proportions of the remote-pumping SFRC with *v*_f_ = 0.4%, 0.8%, and 1.2% (identified as SF0.4, SF0.8, and SF1.2, respectively), and that of the reference concrete (identified as SF0) are summarized in Table 6.

### 2.3. Test Methods

**Figure 3:**
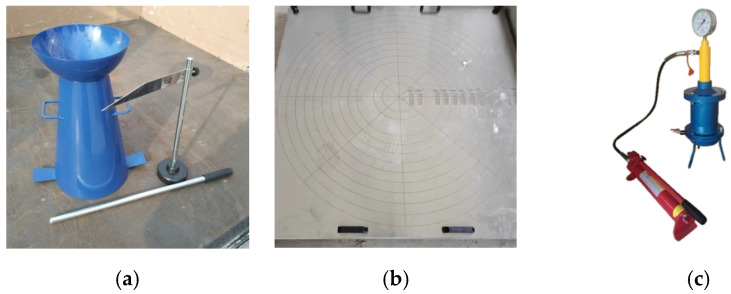
Instruments for testing pumpability: (**a**) slump; (**b**) slump flow; (**c**) pressure bleeding.

The pumpability of the fresh mix was determined according to the specifications of the Chinese code GB/T 50080 [40], and some operation processes were adjusted with reference to a previous study [43]. Some testing instruments are presented in Figure 3. The indices included the slump and slump flow, the static segregation rate, and the pressure bleeding rate. The static segregation rate is the percentage of the mass of segregated binder paste to that of fresh concrete. The segregated binder paste is that flows through a sieve with 5 mm rectangular holes at a static state. The pressure bleeding rate is the ratio of the mass of bleeding water from fresh concrete under pressure for 10 s to that under pressure for 140 s.

**Figure 4:**
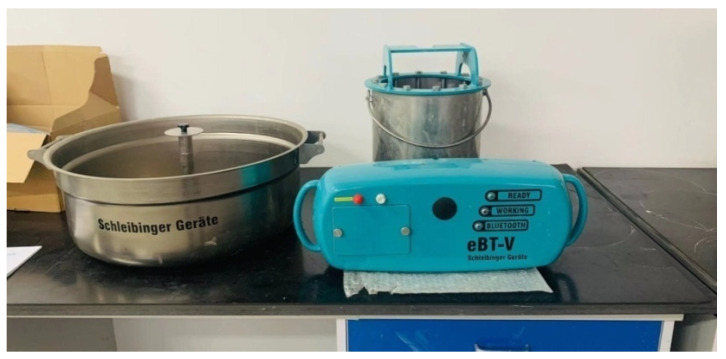
eBT-V concrete rheometer.

The rheological properties of the yield stress and the plastic viscosity of the fresh SFRC and fresh mortar were measured using an eBT-V concrete rheometer produced by Germany Schleibinger CO., Ltd., Buchbach, Germany, as shown in Figure 4. The fresh mortar was wetly sieved from the fresh SFRC using a vibration sieve with a 5 mm mesh size to represent the lubrication layer between the pumping SFRC and the inner surface of the pump pipe due to the similar rheological properties between the lubrication layer and the mortar with almost the same composition [15,18,19].

The cubic compressive strength of the SFRC at curing ages of 7 days and 28 days and the splitting tensile strength, the flexural strength, the impact resistance, and the water impermeability of the SFRC at a curing age of 28 days were measured, as per the specifications of the Chinese codes JG/T 472 [35], GB 50081 [41], SL 352 [44], and GB 50082 [45]. Standard specimens were prepared for the testing. A cubic specimen with dimensions of 150 mm was used for the cubic compressive strength test on a 3000 kN electro-hydraulic servo pressure testing machine and the splitting tensile strength test of the SFRC on a 300 kN electro-hydraulic servo pressure testing machine. A beam specimen of 150 mm × 100 mm × 550 mm was used for the flexural toughness testing of the SFRC, measured by a four-point loading test with a span of 450 mm on a 3000 kN electro-hydraulic servo pressure testing machine. Three specimens were tested as a group. Specimens with a diameter of 150 mm and a thickness of (63 ± 3) mm, six in each group per trial, were tested for the impact resistance testing of the SFRC with an automatic drop-hammer impact tester. Round truncated cone specimens with an upper diameter of 175 mm, a lower diameter of 185 mm, and a height of 150 mm, six in each group per trial, were tested for the water impermeability testing of the SFRC under a 1.2 MPa water pressure for 24 h on a concrete permeability tester.

A single-horizontal-shaft forced concrete mixer was used for mixing the mixes of the SFRC. The crushed limestone and river sand were mixed, which was followed by adding cement and fly-ash, water and the compound pumping agent, and finally by sprinkling steel fibers into the mix. The mix was then stirred for 5 min in total to let the steel fibers become uniformly distributed in the fresh matrix. The fresh mix was poured into standard molds and compacted on a standard vibration table. The top surface was flattened and covered with a thin film for 36 h in a lab room. Then, the specimens were demolded and moved into a standard curing room with a temperature of (20 ± 2) °C and a relative humidity of 95%. In total, 36 cubic specimens, 12 beam specimens, 24 round cake specimens, and 24 round truncated cone specimens were prepared.

## 3. Results of Pumpability

### 3.1. Pumpability

#### 3.1.1. Slump and Slump Flow

As summarized in Table 7 for the test results, the slump and slump flow of the fresh mixes decreased with the increase in the volume fraction of steel fibers. This was due to the steel fibers obstructing the flowing of fresh concrete matrix. Comparatively, the slump and slump flow were slightly influenced by the steel fibers when the volume fraction of steel fiber was less than 0.8%, while they were obviously influenced by the steel fibers when the volume fraction reached 1.2%. The slump loss of the SFRC was 15–35 mm after standing for 1 h, which met the limit of 30 mm specified in the Chinese code GB 50164 [46].

**Figure 5:**
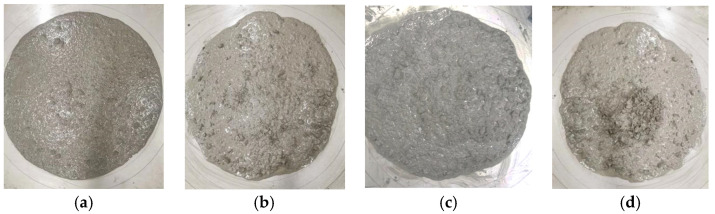
Photos of slump-flow test of SFRC: (**a**) no fiber; (**b**) 0.4% steel fiber; (**c**) 0.8% steel fiber; (**d**) 1.2% steel fiber.

As can be observed in the photos for testing the slump flow of the fresh mixes exhibited in Figure 5, all the slump-flow bodies presented a glossy surface with a well-coated paste for the aggregates and steel fibers, and no water bleeding or aggregate segregation appeared, except for a central bulge that existed on the slump-flow body of the SFRC with a 1.2% volume fraction of steel fiber.

In general, with a proper slump and slump flow, the SFRC with a 0.4–1.2% volume fraction of steel fiber could be long-distance pumped over 1200 m.

#### 3.1.2. Static Segregation and Pressure Bleeding

**Figure 6:**
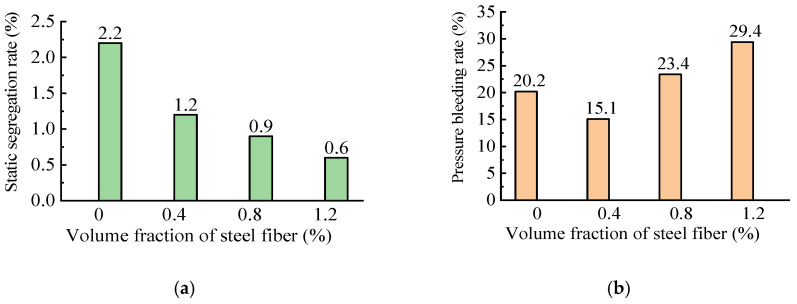
Test results of fresh mixes: (**a**) static segregation rate; (**b**) pressure bleeding rate.

As presented in Figure 6 of the test results, the static segregation rate of the fresh mixes was reduced from 2.2% to 0.6% as the volume fraction of steel fiber increased from zero to 1.2%. This indicated that the steel fibers contributed to the binder paste being retained in the fresh mix due to surface coating and the constituents gathering. In general, the values were far below the limit of 10% for pumped concrete specified in the Chinese code JGJ/T 10 [5].

Meanwhile, compared to the reference concrete, the SFRC with a 0.4% volume fraction of steel fiber had a lower pressure bleeding rate, while the SFRC with a 0.8% and 1.2% volume fraction of steel fiber had a higher pressure bleeding rate. This was attributed to the increased water dosage in the mixing proportion of the SFRC that ensured that the steel fibers were coated with enough binder paste. However, the pressure bleeding rate was much lower compared to that of 36% in the SFRC designed by Jiang et al. [28,29,30] for vertical pumping up to 308 m with a 0.8% volume fraction of steel fiber. The increase in the volume of the binder paste increased the water bleeding possibility of the fresh mix under pressure. However, the experimental pressure bleeding rate was lower than the limit of 40% specified in the Chinese code JGJ/T 10 for pumped concrete [5].

#### 3.1.3. Rheological Properties

**Figure 7:**
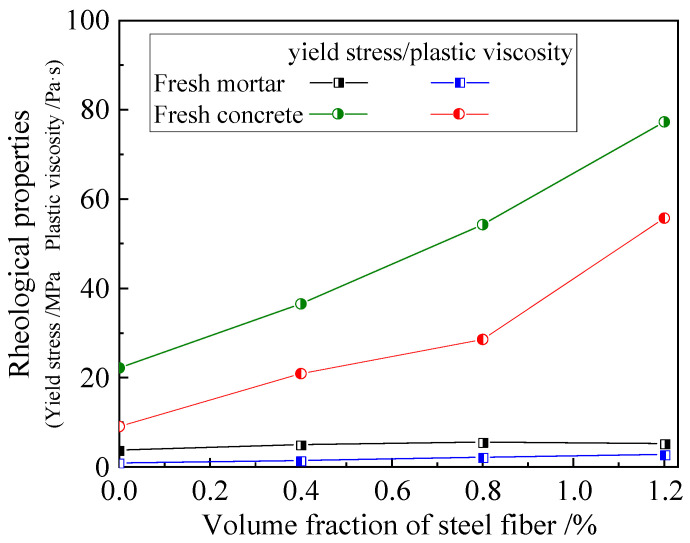
Rheological properties of SFRC and wet-sieving mortar.

As exhibited in Figure 7 for the test results, the yield stress and the plastic viscosity of the fresh mixes were markedly increased with the volume fraction of steel fiber. This indicated that the linkage of the steel fibers across the coarse aggregates disturbed the flow regime of the binder paste and impeded the flow deformation of the binder paste among the gaps of the coarse aggregates. In addition, the yield stress and the plastic viscosity of the fresh mortar were basically kept constant, and these were about 1/6~1/15 and 1/10~1/24 of those of the SFRC, respectively. Similar to that of the pumped concrete [15,18], the mortar with a very low yield stress and plastic viscosity offered a lubrication layer between the fresh SFRC and the inner surface of the pump pipe. This revealed that the volume of the binder paste increased with the volume fraction of the steel fiber and ensured a sufficient coating on the coarse aggregates and steel fibers, which provided the benefit of reducing the pumping pressure with a decreased viscous resistance.

#### 3.1.4. Pumping Test Verification

**Figure 8:**
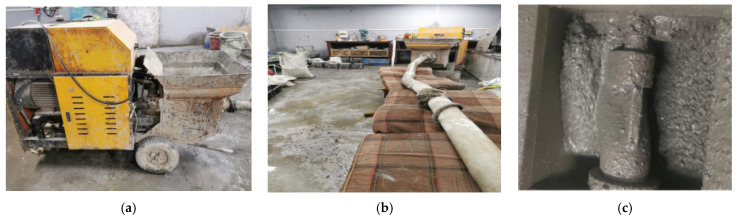
Equipment for pumping test of fresh mix: (**a**) secondary structure pump; (**b**) pump pipe linked to concrete pump; (**c**) fresh mix in the hopper.

To verify the pumpability of the fresh SFRC in practice, a pumping test was conducted in this study with a secondary structure concrete pump. As exhibited in Figure 8, the pump was composed of a hopper, piston cylinder, S-pipe valve, and pump pipe. The fresh mix was automatically pumped by the S-pipe valve under the air pressure of the piston cylinder. The pump capacity was 10 m^3^/h with a rated outlet pump pressure of 8 MPa. The pump pipe was 80 mm in diameter with a length of 6 m.

According to the specifications of the Chinese code JGJ/T 10 [2], the pumping distance can be predicted by formulas as follows,

(4) $$L_{\max}=\frac{P_{c}-P_{f}}{\Delta P_{H}}\times 10^{6}$$

(5) $$\Delta P_{H}=4\left(k_{1}+k_{2}\left(1+t_{1}/t_{2}\right)V\right)\alpha_{1}/D$$

(6) $$V=\frac{Q}{900\pi D^{2}}$$

where *L*_max_ is the maximum pumped distance, m; *P*_c_ is a rated outlet pump pressure, MPa; *P*_f_ is the pressure loss of pump system, which was *P*_f_ = 0.2 MPa in this study; ∆*P*_H_ is the pressure loss per meter of pump pipe, Pa/m; *k*_1_ is the adhesion coefficient of the fresh mix to the pump pipe, *k*_1_ = 300 − *S*_1_, Pa; *k*_2_ is the velocity coefficient of the fresh mix flowing in the pump pipe, *k*_2_ = 400 − *S*_1_, Pa·s/m; *S*_1_ is the slump of the fresh mix, cm; *t*_2_/*t*_1_ is the ratio of the valve switch time to the piston pressure concrete time, which was *t*_2_/*t*_1_ = 0.3 in this study; *α*_1_ is the ratio of the transversal pressure to the axial pressure of the concrete, which was *α*_1_ = 0.9 in this study; *V* is the mean velocity of the fresh mix in the pump pipe, m/s; *Q* is the pump capacity, m^3^/h; and *D* is the diameter of the pump pipe, m.

Therefore, the pumping distances of the fresh mixes were predicted and are listed in Table 8. Tests were conducted to simulate pumping distances of 1200 m and 800 m for the fresh mixes with different volume fractions of steel fiber. The fresh mix was pumped through a 6 m long pump pipe and refilled into the pump hopper to realize a continuous cyclic pumping process. In general, the pumping process was fluent without plugging, and the pumped fresh mixes were cohesive without bleeding and fiber gathering. The test results of the slump and slump flow of the fresh mixes after being pumped are also summarized in Table 8. It can be seen that the slump loss was 30–60 mm, while the slump flow decreased by 90 mm to 160 mm. However, the fresh mix kept a flowability at a high-flowing level with a slump of over 160 mm, thus ensuring the construction quality of the SFRC [38].

### 3.2. Strength

#### 3.2.1. Compressive Strength

**Figure 9:**
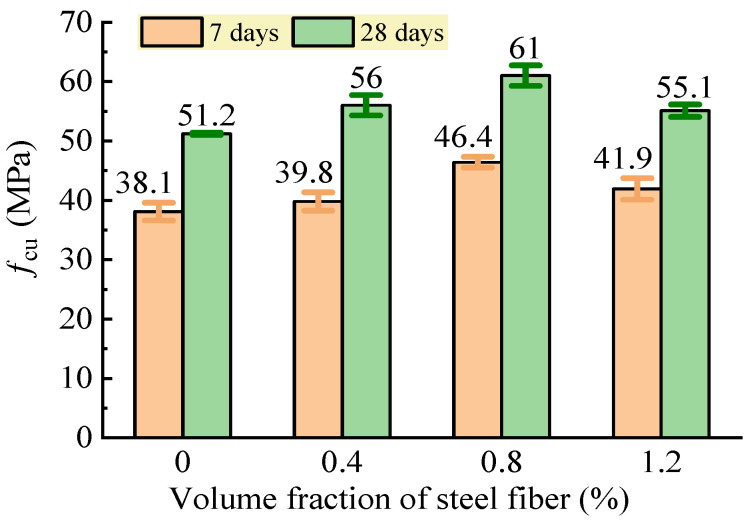
Changes in cubic compressive strength of SFRC.

The test results of the cubic compressive strength of the SFRC are shown in Figure 9. Compared to the reference concrete without steel fiber, the SFRC had an increased cubic compressive strength that reached a maximum with a volume fraction of steel fiber at 0.8%. The increment at a curing age of 7 days was 4.5%, 21.8%, and 10% for the SFRC with a 0.4%, 0.8%, and 1.2% volume fraction of steel fiber, respectively, while that at a curing age of 28 days was 9.4%, 19.1%, and 9.6%, respectively. This indicated that the random distribution of the steel fibers reinforced the concrete matrix with reliable bonding and a high modulus of elasticity that could provide a strong confinement to the transversal deformation of the cubic specimens under compression. Due to the distribution of the steel fibers changing due to multiple factors, including the constituent and mixing proportion of the concrete matrix, the mixing proportion adjusted with the water dosage and the sand ratio by using Formulas (1) and (2) influenced the reinforcing effectiveness with the volume fraction of steel fiber. Therefore, compared with that of the reference concrete, an increase in the compressive strength was presented with an increase in the volume fraction of steel fiber from 0.4% to 1.2%, while a peak was reached at a 0.8% volume fraction of steel fiber.

#### 3.2.2. Splitting Tensile Strength

**Figure 10:**
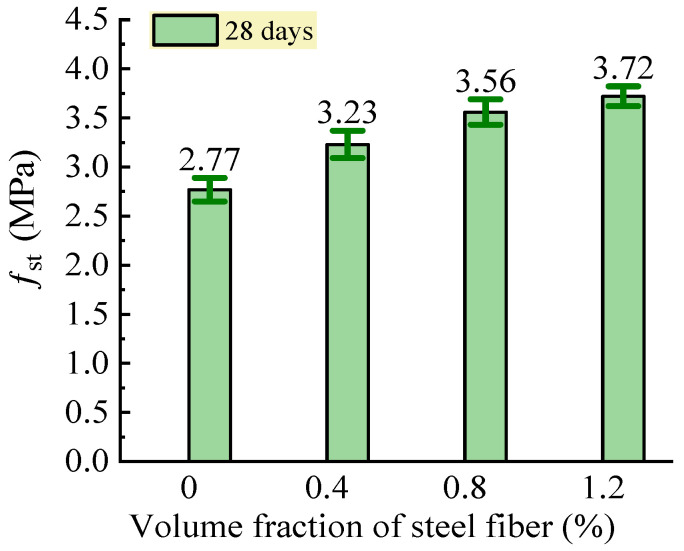
Changes in splitting tensile strength of SFRC.

The test results of the splitting tensile strength of the SFRC are shown in Figure 10. This presented an obvious increasing relationship between the splitting tensile strength and the volume fraction of steel fiber. Compared to the reference concrete without steel fiber, the SFRC had a splitting tensile strength that increased by 16.6%, 28.5%, and 34.3% with a volume fraction of steel fiber of 0.4%, 0.8%, and 1.2%, respectively. Therefore, the bridging effect of the steel fibers across the micro-defects in the internal concrete matrix before loading and the macro-cracks under load increased when more steel fibers were distributed in the concrete matrix due to the increase in the volume fraction of steel fiber [24,25]. This effectively reinforced the tensile properties of the constituent composites of the SFRC.

With reference to the specifications of the Chinese code JG/T 472 [35], a prediction equation was obtained by the linear fitting analysis of the test data, which was:

(7) $$f_{\mathrm{ft}}=f_{t0}\left(1+0.765F\right)$$

where *f*_f0_ is the splitting tensile strength of the reference concrete with the same matrix as the SFRC. *f*_f0_ = 2.84 MPa according to the fitting analysis, and it was 2.77 MPa in the experimental results of this study.

A good agreement between the experimental and the predicted results was obtained with a Pearson’s ratio of 0.978. The reinforcing coefficient of the steel fiber on the tensile strength obtained in this study was 0.765, which was almost equal to the value of 0.778 specified in the Chinese code JG/T472 [35]. This means that the tensile strength of the remote-pumping SFRC could be predicted with the same equation as conventional SFRC.

#### 3.2.3. Flexural Strength

**Figure 11:**
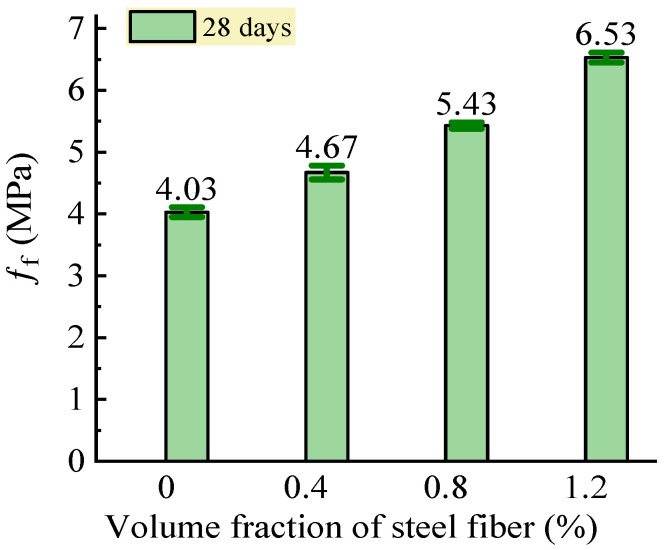
Changes in flexural strength of SFRC.

The test results of the flexural strength of the SFRC are shown in Figure 11. This presented a remarkable increase in the flexural strength with the increase of the volume fraction of steel fiber. Compared to the reference concrete without steel fiber, the flexural strength of the SFRC increased by 15.9%, 34.7%, and 62.0% with a volume fraction of steel fiber of 0.4%, 0.8%, and 1.2%, respectively. With referenced to the specifications of the Chinese code JG/T 472 [35], a prediction equation was obtained by the linear fitting analysis of the test data, which was:

(8) $$f_{\mathrm{ftm}}=f_{\mathrm{tm}0}\left(1+1.44F\right)$$

where *f*_fm0_ is the flexural strength of the reference concrete with the same matrix as the SFRC. *f*_fm0_ = 3.93 MPa according to the fitting analysis, and it was 4.03 MPa in the experimental results of this study.

A good agreement between the experimental and the predicted results of the flexural strength of the SFRC was obtained with a Pearson’s ratio of 0.986. The reinforcing coefficient of 1.44 obtained in this study was higher than the value of 1.02 specified in the Chinese code JG/T47 [35]. This means that the remote-pumping SFRC had a higher flexural strength than conventional SFRC. The reason was mainly due to the orientation tendency of the steel fibers along the longitudinal direction of the beam specimens, which was due to the steel fibers being distributed with the flowing of the fresh mix. This made the longitudinally distributed steel fibers withstand the tension across the cracks with a stronger bond strength [23,24,25]. In addition, with the increase in the volume fraction of steel fiber, more steel fibers across the cracks in the pure bending segment of the beam specimens built a stronger bridge that resisted the tensile stress transferred from the cracked concrete matrix [24].

### 3.3. Impact Resistance

**Figure 12:**
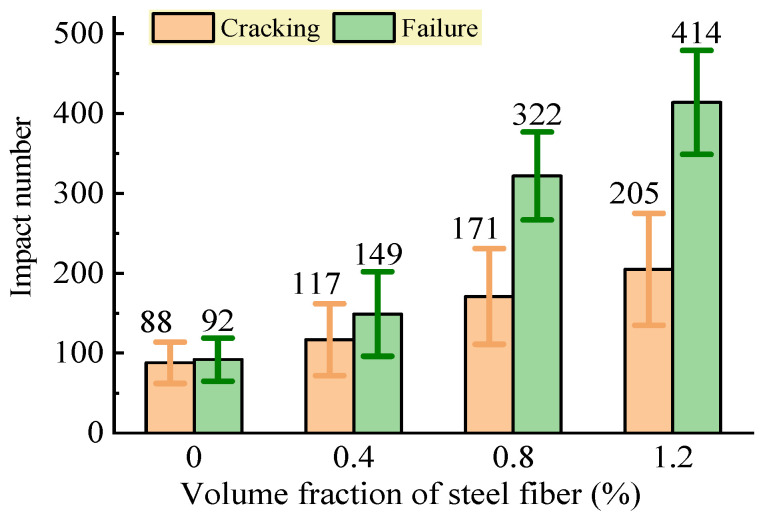
Changes in impact resistance of SFRC.

The test results of the impact number of the SFRC at the initial cracking and the failure states are shown in Figure 12. This shows that the remote-pumping SFRC had an obviously increased impact resistance with the increase in the volume fraction of steel fiber that could absorb the kinetic energy under repeated impaction. Compared to that of the reference concrete without steel fiber, the impact number at the initial cracking of the remote-pumping SFRC with a 0.4%, 0.8%, and 1.2% volume fraction of steel fiber was increased by 33.0%, 94.3%, and 133.0%, respectively, and that at failure increased by 62.0%, 250.0%, and 350.0%, respectively. This was attributed to the framework of the steel fibers in the concrete matrix that postponed the extension of the cracks and enabled the entirety of the SFRC to resist the repeated impaction. In addition, the linkage of the steel fibers in the cracks of the concrete matrix provided the benefit of dispersing the stress concentration at the ends of the cracks, thus postponing the crack extension, and the contributed to absorbing the kinetic energy. It was noticed that the discreteness of the test data for the six specimens in the trial increased with the volume fraction of steel fiber. This revealed that the impact resistance of the SFRC highly depended on the distribution of the steel fibers in the concrete matrix.

**Figure 13:**
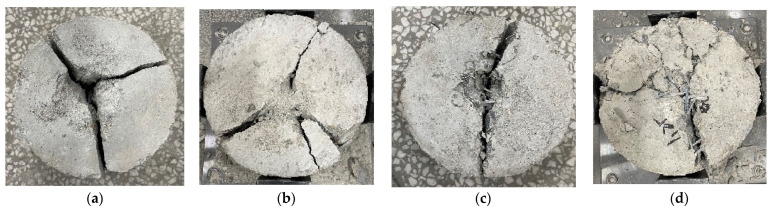
Impact failure patterns of tested specimens: (**a**) no fiber; (**b**) 0.4% steel fiber; (**c**) 0.8% steel fiber; (**d**) 1.2% steel fiber.

As exhibited in Figure 13, different failure patterns appeared in the tested specimens. The concrete specimens were divided into three blocks by straight cracks with a larger and deeper crushed impact pit. This was improved by adding a 0.4% volume fraction of steel fiber, although a similar failure pattern still appeared. For the remote-pumping SFRC with a 0.8% volume fraction of steel fiber, the failure pattern changed into two integral blocks divided by a crack that was linked by steel fibers, and the impact pit became smaller. When the volume fraction of steel fiber increased to 1.2%, the entirety of the SFRC specimens had a markedly improved resistance to the repeated impaction, and the failure pattern changed into several cracked blocks that were linked together by the steel fibers.

### 3.4. Water Permeability

The test results of the permeable height of water into the SFRC are summarized in Table 9. The results showed an increased tendency of the permeable height of water into the SFRC with the increase of the volume fraction of steel fiber. Compared to that of the reference concrete without steel fiber, the permeable height of water into the SFRC increased by 15.4%, 19.8%, and 59.6% with a 0.4%, 0.8%, and 1.2% volume fraction of steel fiber, respectively. In addition, the deviation of the test results also tended to increase with the volume fraction of steel fiber. This was related to the adjusting of the mixing proportion of the remote-pumping SFRC with a higher water dosage and sand ratio due to the increase in the volume fraction of steel fiber. The number of micro-pores and defects in the concrete matrix increased due to a more residual free water, which provided a penetrative possibility by steel fibers. This increased the potential paths for water penetration along the weak interfaces between the steel fibers and the concrete matrix. According to the specifications of the Chinese code SL352 [44], the relative permeability coefficient *k*_r_ can be calculated as follows:

(9) $$k_{r}=0.03h_{m}^{2}/2TH$$

where *h*_m_ is the average permeable height of water, cm; *T* is the sustainable time, h; and *H* is the pressure presented by the water head, cm.

## 4. Discussion

The optimum mixing proportions of the reference concrete designed using the absolute volume method was effective for determining the key factors that determined the pumpability and compressive strength. This provided the basis for the design of the mixing proportion of the SFRC by only adjusting the main factors, including the water dosage and the sand ratio with the volume fraction of steel fiber. This ensured the similar rheological properties of the lubrication layer between the fresh SFRC and the interface of the pump pipe during the pumping process. Therefore, the pumpability of the fresh SFRC could meet the specifications for remote-pumping construction. The water-to-binder ratio of the SFRC was consistent with that of the reference concrete to remain a reliable compressive strength.

With the increase in the volume fraction of steel fiber, the static segregation rate of the fresh SFRC obviously decreased and was far below the limit of 10% for pumped concrete specified in the Chinese code JGJ/T 10 [5]. This was attributed to the linkage of the steel fibers to the concrete matrix, which confined the separation of the binder paste from the fresh mix. Relatively, the pressure bleeding rate of the fresh SFRC increased with the volume fraction of steel fiber and had a tendency to reach the limit of the specifications. Therefore, the static segregation test was unnecessary when the pressure bleeding rate met the specification.

The splitting tensile strength of the remote-pumping SFRC was similar to that predicted with the specified reinforcing coefficient of steel fiber for conventional SFRC. The flexural strength of the remote-pumping SFRC was much higher than that predicted using the specified reinforcing coefficient of steel fiber for conventional SFRC. This revealed that, similar to that along the lowing direction of flowable SFRC [24], an obvious distribution of the steel fibers along the longitudinal direction of the beam specimens contributed to the specimens withstanding the tensile stress under bending. Therefore, the tensile strength of the remote-pumping SFRC depended on the forming method with different sizes of specimens.

The dispersion of the impact resistance of the SFRC increased with the volume fraction of steel fiber, although the failure pattern of the SFRC specimens was improved from the separated three blocks to the cracked parts linked by the steel fibers. This further proves that the uniform distribution of steel fibers plays an important role in ensuring the stable performance of SFRC.

When the volume fraction of steel fiber increased from 0.4% to 1.2%, an increased tendency of the permeable height of water into the SFRC was found with an increased relative permeability coefficient. This is inconsistent with that the SFRC is beneficial to protect steel reinforcements. Therefore, the degree of its influence should be evaluated in the future.

## 5. Conclusions

Concerning the construction performance and the mechanical properties of remote-pumping SFRC, an experimental study was conducted in this paper. The conclusions can be briefly summarized as follows:

(1) The mixing proportion of the remote-pumping SFRC could be designed based on that of reference concrete by adjusting the water dosage and the sand ratio with the volume fraction of steel fiber. The pumpability of the fresh SFRC could be represented by indices of the slump flowability and the pressure bleeding rate. When the pressure bleeding rate met the limits of the specifications, the static segregation rate was unnecessary for the SFRC.

(2) When the volume fraction of steel fiber varied from 0.4% to 1.2%, the remote-pumping SFRC had a slightly increased cubic compressive strength than and a similar splitting tensile strength to that predicted using the same reinforcing coefficient of steel fiber for conventional SFRC. The remote-pumping SFRC presented a flexural strength with a much higher reinforcing coefficient of steel fiber of 1.44 compared to the value of 1.02 specified for conventional SFRC.

(3) When the volume fraction of steel fiber was increased from 0.4% to 1.2%, the entirety of the SFRC specimens had a markedly improved resistance to repeated impaction, and the failure pattern changed to being cracked into several blocks that were linked together with the steel fibers. An acceptable water impermeability was obtained for the remote-pumping SFRC.

(4) Due to the increase in the sand ratio and the water dosage with the volume fraction of the steel fiber, which was carried out in order to maintain a reasonable pumpability of the fresh SFRC, the shrinkage and early cracking resistance of remote-pumping SFRC should be examined in future studies.

## Figures and Tables

**Table 1:** Physical and mechanical properties of cement.

Apparent Density (kg/m^3^)	Fineness (m^2^/kg)	Water Requirement of Normal Consistency (%)	Setting Time (min)		Compressive Strength (MPa)		Flexural Strength (MPa)	
			Initial	Final	3 d	28 d	3 d	28 d
3052	356	26.0	223	273	26.7	47.45	4.7	12.8

**Table 2:** Physical and mechanical properties of fly-ash.

Apparent Density (kg/m^3^)	Specific Surface Area (m^2^/kg)	Fineness (%)	Water Demand (%)	Water Content (%)	Activity Index (%)
2250	406	20.3	98	0.42	86.0

**Table 3:** Chemical compositions of binders including cement and fly-ash.

Binder	Chemical Composites (%)						
	CaO	MgO	Al_2_O_3_	Fe_2_O_3_	SiO_2_	SO_3_	LOI
Cement	62.33	2.33	5.31	3.37	19.72	3.33	2.68
Fly-ash	0.26	3.80	31.14	4.16	50.26	2.16	2.34

**Table 4:** Main properties of coarse and fine aggregates.

Aggregates	Density (kg/m^3^)			Porosity (%)		Water Content (%)	Water Absorption (%)	Mud Content (%)
	Apparent	Bulk	Closed Packing	Bulk	Closed Packing			
Crushed limestone	2730	1505	1700	44.9	37.8	0.31	0.75	2.70
River sand	2597	1600	1685	38.4	35.1	0.75	1.13	1.90

**Table 5:** Flowability of concrete with different pumping heights/distances.

Pumping Height/Distance (m)	200/600	400/1200	>400/1200
Slump (mm)	190~220	230~260	-
Slump flow (mm)	-	450~590	600~740

**Table 6:** Mixing proportions of SFRC and reference concrete.

Identifer	*v*_f_ (%)	Dosage of Raw Materials (kg/m^3^)						
		Cement	Fly-Ash	Water	Crushed Limestone	River Sand	Steel Fiber	Pumping Agent
SF0	0	356.3	152.7	178.1	834.8	834.8	0	7.1
SF0.4	0.4	370.5	158.8	185.3	818.7	850.1	31.4	7.6
SF0.8	0.8	384.7	168.9	192.4	802.6	865.4	62.8	8.2
SF1.2	1.2	399.0	171.0	199.5	786.5	880.7	94.2	8.7

**Table 7:** Test results of slump and slump flow of fresh mixes.

Fresh Mix	Slump (mm)			Slump Loss at 1 h (mm)	Slump Flow (mm)		
	Initial	Stand for 0.5 h	Stand for 1 h		Initial	Stand for 0.5 h	Stand for 1 h
SF0	270	270	255	15	700	680	650
SF0.4	260	250	250	10	680	660	625
SF0.8	250	235	225	25	640	610	585
SF1.2	240	220	210	30	560	515	485

**Table 8:** Pumping test results of fresh mixes with different volume fractions of steel fiber.

Identifer	*V* (m/s)	*S*_1_ (mm)	∆*P*_H_ (Pa/m)	*L*_max_ (m)	Simulated Distance (m)	Pumping Time (min)	After Pumping (mm)		Loss Compared to That before Pumping (mm)	
							Slump	Slump Flow	Slump	Slump Flow
SF0	0.553	270	5555	1224	1200	36	240	550	30	150
SF0.4	0.553	265	6329	1074	1200	36	200	520	60	160
SF0.8	0.553	260	7102	957	800	24	210	550	40	90
SF1.2	0.553	250	8262	822	800	24	195	470	45	90

**Table 9:** Test results of water permeability of SFRC.

Specimen	Permeable Height of Water (cm)		Relatively Permeability Coefficient (10^−7^ cm/h)
	Average	Deviation	
SF0	1.36	0.29	0.944
SF0.4	1.57	0.39	1.258
SF0.8	1.63	0.30	1.357
SF1.2	2.17	0.37	2.404

## Data Availability

Data are available as request.
